# Fast and sensitive validation of fusion transcripts in whole-genome sequencing data

**DOI:** 10.1186/s12859-023-05489-5

**Published:** 2023-09-23

**Authors:** Völundur Hafstað, Jari Häkkinen, Helena Persson

**Affiliations:** Faculty of Medicine, Department of Clinical Sciences Lund, Oncology, Lund University Cancer Centre, Lund, Sweden

**Keywords:** Gene fusion, Fusion transcript, RNA sequencing, Whole-genome sequencing, Breast cancer

## Abstract

**Background:**

In cancer, genomic rearrangements can create fusion genes that either combine protein-coding sequences from two different partner genes or place one gene under the control of the promoter of another gene. These fusion genes can act as oncogenic drivers in tumor development and several fusions involving kinases have been successfully exploited as drug targets. Expressed fusions can be identified in RNA sequencing (RNA-Seq) data, but fusion prediction software often has a high fraction of false positive fusion transcript predictions. This is problematic for both research and clinical applications.

**Results:**

We describe a method for validation of fusion transcripts detected by RNA-Seq in matched whole-genome sequencing (WGS) data. Our pipeline uses discordant read pairs to identify supported fusion events and analyzes soft-clipped read alignments to determine genomic breakpoints. We have tested it on matched RNA-Seq and WGS data for both tumors and cancer cell lines and show that it can be used to validate both new predicted gene fusions and experimentally validated fusion events. It was considerably faster and more sensitive than using BreakDancer and Manta, software that is instead designed to detect many different types of structural variants on a genome-wide scale.

**Conclusions:**

We have developed a fast and very sensitive pipeline for validation of gene fusions detected by RNA-Seq in matched WGS data. It can be used to identify high-quality gene fusions for further bioinformatic and experimental studies, including validation of genomic breakpoints and studies of the mechanisms that generate fusions. In a clinical setting, it could help find expressed gene fusions for personalized therapy.

**Supplementary Information:**

The online version contains supplementary material available at 10.1186/s12859-023-05489-5.

## Background

Fusion genes are created when two separate genes are merged as a result of a chromosomal rearrangement. This can lead to the formation of a chimeric gene that combines functional domains from both fusion partner genes, or to a promoter swapping event, where the promoter of one gene is replaced with another, leading to altered gene expression. In cancer, gene fusions occur frequently due to the genetic instability of cancer cells. The cancer-specific nature of gene fusions has rendered them attractive targets for cancer therapy. Inhibitors that disrupt the activity of the fusion proteins generated by in-frame gene fusions have shown promise in treating cancers that harbor such fusions [1–6]. Gene fusions can also serve as cancer-specific biomarkers [7, 8]. The detection of gene fusions in tumor tissue, circulating tumor cells or cell-free DNA can aid in cancer diagnosis, prognosis, and personalized treatment [9]. Gene fusions can help identify patients who are likely to respond to targeted therapies, allowing selection of the most appropriate treatment option for each patient.

Fusions can impact the cell in several ways. In-frame fusions that produce chimeric proteins often drive cancer development and progression via dysregulation of signaling pathways related to the fusion genes. Promoter-swapping events involving oncogenes or tumor suppressors can lead to the upregulation of oncogenic activity and the downregulation of tumor-suppressive activity without changing the protein-coding sequence of the genes involved, also resulting in cancer progression [10, 11]. We have previously identified an additional function for gene fusions, by showing that they can impact the expression of non-coding RNAs (ncRNAs) that are located in the introns of fusion genes, and that ncRNA host genes are over-represented in fusion events [12–14]. However, not all gene fusions are equal in their impact on cancer cells, and many fusions detected in tumors are likely inactive passenger events [15].

Gene fusions are commonly detected in RNA sequencing (RNA-Seq) data in the form of chimeric fusion transcripts. Many software exist to detect fusion transcripts, but the overlap of detected fusion events tends to be small and the tools output an unknown number of false positives [16, 17]. If gene fusions are to be exploited for therapy, methods to accurately detect true events are imperative. Whole-genome sequencing (WGS) has the advantage of being able to detect structural variants at a genomic scale, but it is challenging to assess whether a fusion event found purely at the DNA level has the potential to be processed into a functional transcript. Another advantage of WGS is that exact genomic breakpoints can often be identified. This allows for experimental validation in genomic DNA and analyses of the genetic mechanisms behind fusion generation. True positive fusion transcripts are here defined as fusions that can be detected at both RNA and DNA level in the analyzed sequencing data. This is likely to result in identification of high-confidence fusion events, although detection is limited by sequencing data quality and depth.

Here we have combined the strengths of both RNA-Seq and WGS data and developed a pipeline to validate gene fusions found in RNA-Seq data at the WGS level. The pipeline consists of extracting, processing and filtering discordant read pairs from specific areas of the genome defined by the detected fusion junctions of fusion transcripts. If discordant read pairs are detected, we also attempt to locate the genomic breakpoints of the fusion partners. By using information on the fusion junctions, we can drastically limit the regions in the genome where we search for fusion support. This approach lowers the number of false positives and allows us to use very sensitive filtering criteria. Since our pipeline only uses the coordinates of annotated genes and fusion junctions, it can be applied to the output of any software for detection of fusion transcripts.

As proof of concept, we applied our pipeline to a diverse range of samples that have matched RNA-Seq and WGS data from the breast invasive carcinoma (BRCA), glioblastoma multiforme (GBM), diffuse large B-cell lymphoma (DLBC), and acute myeloid leukemia (LAML) cohorts in The Cancer Genome Atlas (TCGA). We also used published information on experimentally validated gene fusions in eight cancer cell lines to evaluate our pipeline. As part of the evaluation process, we compared our pipeline to two established tools for detecting structural variants in WGS data: Manta and BreakDancer. Our tool proved to be very sensitive, being able to validate more fusion events in both the clinical samples and cell lines compared to the other tools. In addition, as our pipeline utilizes a focused search based on the results of fusion transcript calling, it runs substantially faster and requires much less computing power than tools designed to query the whole genome. In summary, our pipeline provides a novel and sensitive approach to validate fusion transcripts in samples with matched WGS data. By using our pipeline to validate fusion transcripts at the DNA level, future studies on gene fusions can be limited to only high-confidence events. This can save time and resources that would have been otherwise spent on analyzing false positives and will ultimately lead to more accurate conclusions about the roles of gene fusions in cancer.

## Implementation

**Fig. 1:**
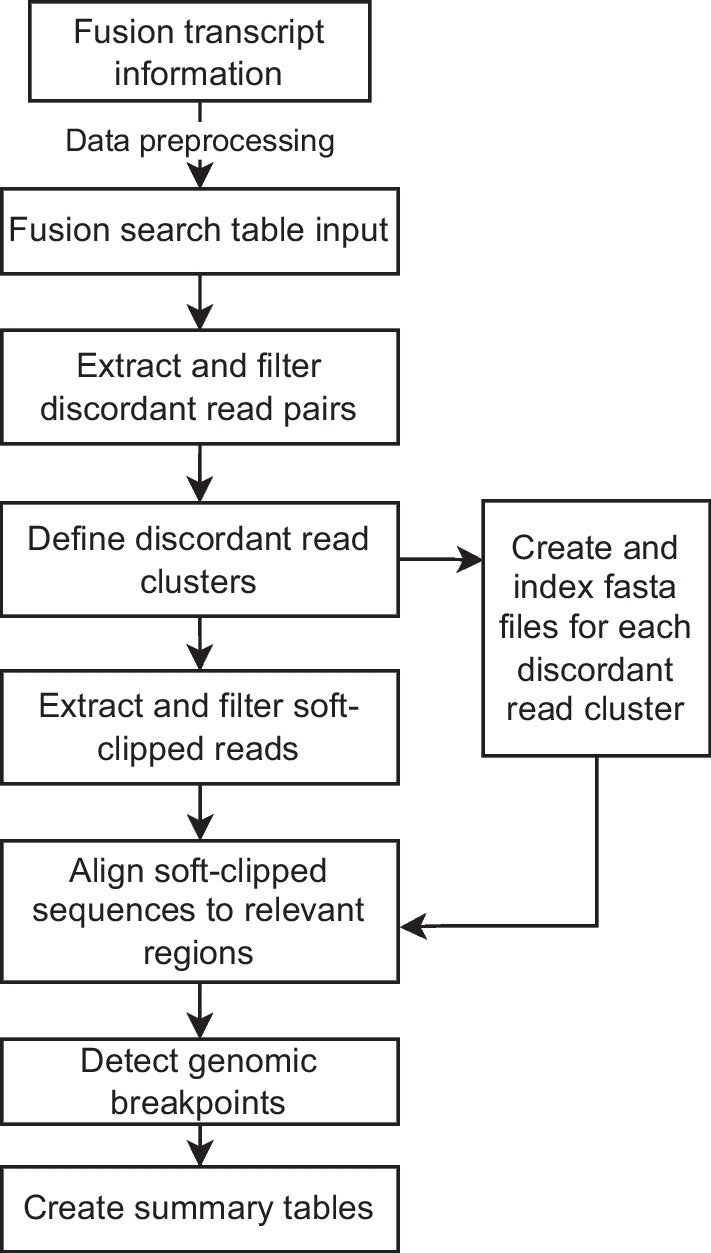
Schematic overview of the fusion validation pipeline. In brief, processed fusion-transcript information is used as input to search for discordant read pairs in matched WGS data. If discordant read pairs are found, reads with high-quality soft-clipped sequences are extracted and aligned to the other fusion partner in order to detect genomic breakpoints

Our pipeline consists of a series of scripts that attempt to validate putative fusion transcripts on the DNA level by querying and processing WGS data. If evidence is found for a fusion, we also attempt to identify genomic breakpoints that support the fusion for both fusion gene partners. An overview of the pipeline can be seen in Fig. 1.

**Fig. 2:**
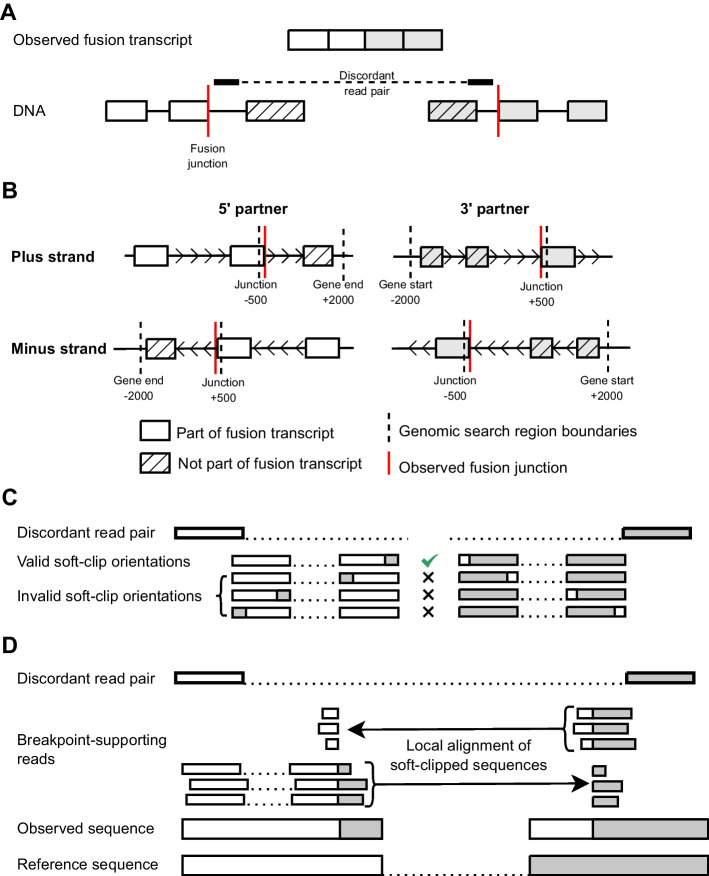
The regions to search for discordant read pairs are defined by the junction coordinates of the observed fusion transcript. Genomic evidence for a fusion will theoretically be found downstream of the sequences observed on fusion transcript for the 5′ partner and upstream for the 3′ partner, thereby limiting the region needed to search for discordant reads (**A**). Based on the strand that the fusion partner is located on, the defined search regions change (**B**). Reads supporting a genomic breakpoint must have a soft-clipped end in an “outie” orientation on the outer read of the pair with respect to the discordant read location (**C**). Once discordant read pairs supporting a fusion transcript have been identified, we attempt to identify reads with high quality soft-clipped ends that support a genomic breakpoint. Soft-clipped sequences are locally aligned close to the discordant read mate. Note that in this example, each genomic breakpoint is supported by six distinct reads (**D**)

Based on the reported fusion junction coordinates, we define search regions in the genome for each fusion partner. These are the regions of the genome that will be queried for discordant read pairs that support the observed fusion transcript. For 5′ partners the search region spans from the fusion junction coordinate to the end of the gene, and for the 3′ partners it spans from the start of the gene to the fusion junction coordinate (Fig. 2A). To ensure comprehensive detection of fusion breakpoints, each search region is padded with 500 base pairs (bp) on the side of the fusion junction and 2 kilobase pairs (kb) up- or downstream of the start or end of the gene, respectively (Fig. 2B). It is important to note that the “start” of each search region is defined in genomic coordinate space, so partners on the minus strand must have their start and end switched accordingly. Prior to running the pipeline, each sample and fusion transcript should be given unique identifiers to facilitate downstream analysis.

The files required to run the pipeline consists of aligned WGS data in BAM format, the corresponding reference genome in fasta format, and a tab-separated table with information on the fusion transcripts that are to be validated. The input table should have the columns listed in Table 1.

**Table 1:** Input table format required for the WGS fusion validation pipeline

Input column	Description	Example
fusion_id	Unique identifier for each fusion transcript	1
sample_id	Unique identifier/barcode for each sample	sample_01
path	Full path to the raw WGS files	~/raw_data/sample1/sample1.bam
fiveprime_chr	5′ chromosome	8
fiveprime_strand	5′ strand	-
fiveprime_junction	5′ fusion junction coordinate	55,013,468
fiveprime_search_start	5′ fusion search region start coordinate	54,956,927
fiveprime_search_end	5′ fusion search region end coordinate	55,013,968
threeprime_chr	3′ chromosome	8
threeprime_strand	3′ strand	+
threeprime_junction	3′ fusion junction coordinate	61,531,139
threeprime_search_start	3′ fusion search region start coordinate	61,427,495
threeprime_search_end	3′ fusion search region end coordinate	61,531,639

The first step in the pipeline is to iterate over each fusion event in the input table and extract discordant reads that potentially support the fusion transcript using SAMtools [18]. Reads from the 5′ search region are extracted and kept if the mate maps to the 3′ search region, and vice versa. In the case of adjacent genes, the search regions may overlap and result in reads being extracted that are consistent with normal fragment sizes. Intrachromosomal read pairs corresponding to fragments of 4 kb or smaller are therefore discarded, since these are unlikely to be derived from real gene fusion events. This cutoff was selected based on the fragment size distribution but can be changed by the user (see Additional file 1: Figure S1A). Reads labeled as PCR/optical duplicates or with a mapping quality of 0 (multimapping reads) are discarded. Both reads in a pair are discarded if either one fails to pass the filtering. The read pairs remaining can be considered to support the associated gene fusions and are saved in an output table which also contains information from SAMtools and the associated fusion transcript.

After identifying discordant read pairs that pass filtering, the pipeline attempts to locate genomic breakpoints associated with the fusion event. Breakpoints are identified from reads that are located close to a discordant read and that have soft-clipped ends, where the soft-clipped sequence aligns in a region close to the mate of the discordant read. Because genomic breakpoints can theoretically only occur within one fragment length of a discordant read, we define a search region for each discordant read identified. This search region extends from the coordinates of the discordant read, with additional padding of 500 bp in the direction of the breakpoint and 50 bp in the opposite direction. In cases where multiple discordant reads support the same fusion event and their respective breakpoint search regions overlap, they are combined into a single search region to avoid fetching the same reads multiple times. Each breakpoint search region can therefore be associated with an arbitrary number of discordant read pairs, so a breakpoint can be linked back to a specific discordant read. The breakpoint search regions are then compiled into a new tab-separated search table, and every read containing a soft-clipped end is subsequently extracted from these defined regions and subjected to rigorous filtering. As before, reads flagged as PCR/optical duplicates are excluded, as well as reads with a mapping quality score of 0. Soft-clipped ends that are shorter than 6 bp or have an average sequencing quality score of 15 or less are discarded as a final filtering step.

The location of a breakpoint-supporting soft-clipped sequence within a read is determined by the fusion partner and the strand it is located on, with only one possible side for each partner (Fig. 2C). Therefore, only reads with soft-clipped ends on the correct side are kept. In order for a soft-clipped read to support a genomic breakpoint, the soft-clipped sequence should also align close to the discordant read mate in the other fusion partner. We therefore align the soft-clipped sequences that pass filtering to the corresponding breakpoint search region in the other fusion partner using Novoalign V3.09.04 (Fig. 2D).

### Sequencing data

WGS and RNA-Seq data for the breast cancer cell lines BT-474 and MCF7 were obtained from the Cancer Cell Line Encyclopedia [19] and downloaded from the Sequence Read Archive, BioProject PRJNA523380, runs SRR8639205, (BT-474 WGS), SRR8652105 (MCF7 WGS), SRR8616195 (BT-474 RNA-Seq) and SRR8615758 (MCF7 RNA-Seq). WGS and RNA-Seq data for the hematological cell lines LAMA-84 (WGS: SRR8652091, RNA-Seq: SRR8615684), MOLM-13 (WGS: SRR17524983, RNA-Seq: SRR8616069), MOLT-4 (WGS: SRR4009283, RNA-Seq: SRR6755970), MV4-11 (WGS: SRR8652133, RNA-Seq: SRR8615687), CCRF-CEM (WGS: SRR4009291, RNA-Seq: SRR6756016), and THP-1 (WGS: SRR8670675, RNA-Seq: SRR8616091) were obtained from the Sequence Read Archive. WGS data for cell lines were downloaded in fastq format and aligned to the human reference genome GRCh38.p14 using BWA v0.7.17. WGS and RNA-Seq data from BRCA, GBM, DLBC, and LAML patient samples were obtained from TCGA.

## Results

### Our WGS validation pipeline confirms the presence of gene fusions

We first assessed the performance of our pipeline for validation of fusion transcripts in BRCA and GBM samples from TCGA. We used FusionCatcher v1.00 [20] to identify fusion transcripts in RNA-Seq data and filtered them based on a criterion of having at least three unique spanning read pairs and zero common mapping reads to reduce the number of false positives. From this filtered list, we selected samples to represent every tenth percentile in number of fusions, resulting in 11 samples for each cohort.

**Fig. 3:**
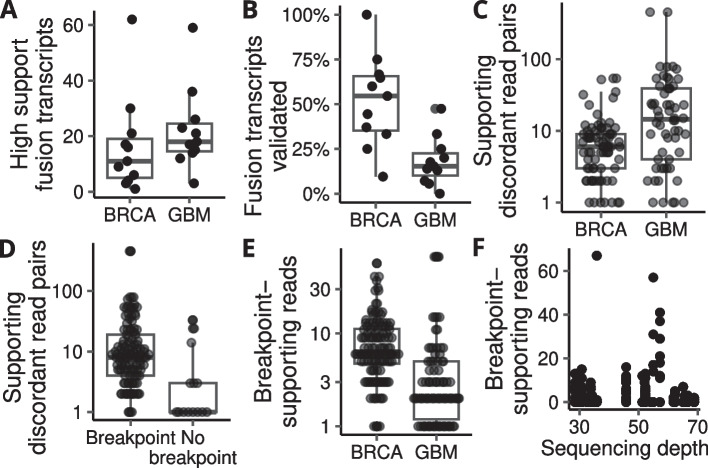
Highly supported fusion transcripts in samples from the TCGA BRCA and GBM cohorts (**A**). A higher fraction of fusion transcripts was validated in the BRCA cohort (**B**), but fusion events in GBM were supported by a higher number of discordant read pairs on average (**C**). Most fusion events were further supported by an identified genomic breakpoint in close proximity to the discordant read pairs (**D**). BRCA had on average more reads supporting a genomic breakpoint (**E**), but the number of breakpoint-supporting reads did not correlate with the sequencing depth of the samples (**F**)

The number of fusion transcripts ranged from 1 to 62 and from 3 to 59 for the BRCA and GBM cohorts, respectively, with means of 16.5 and 22.5 detected fusion transcripts per sample (Fig. 3A). We used information on these fusions to run our pipeline and evaluated the performance by examining the percentage of the fusions validated in each sample. Despite detecting fewer highly supported fusion transcripts in the BRCA cohort, we were able to validate a higher percentage of them on average when compared to GBM. The mean percent of validated fusions per sample was 52% and 18%, respectively (Fig. 3B). Validated fusions in both cohorts generally had a high level of support at the DNA level (≥ 5 supporting discordant read pairs and/or an identified genomic breakpoint), with BRCA having an average of 9 discordant read pairs per fusion with a standard deviation of 11, and GBM fusions having an average of 37, with a standard deviation of 86 (Fig. 3C). Notably, approximately 90% of all fusions validated by a discordant read pair were further supported by an identified genomic breakpoint, and breakpoint identification was positively correlated with the number of discordant read pairs supporting the fusion (r = 0.32, *p* = 1.5 × 10^–4^, Pearson’s product-moment correlation) (Fig. 3D). Despite having fewer discordant read pairs supporting each fusion on average, BRCA had more reads supporting genomic breakpoints compared to GBM (Fig. 3E). Surprisingly, we observed no correlation between the number of reads supporting a genomic breakpoint and the sequencing depth of the sample (Fig. 3F). Overall, fusions that were validated by our pipeline generally had a high level of support, both in the form of discordant read pairs and breakpoint supporting reads. A list of all validated fusions in the TCGA BRCA and GBM samples is included in Additional file 2.

BRCA and GBM tumors can contain several gene fusion events, but it is not clear how many of these represent tumor drivers. For comparison, we therefore also analyzed samples from two TCGA cohorts with hematological malignancies, diffuse large B-cell lymphoma (DLBC) and adult acute myeloid leukemia (LAML), where fusion transcript predictions from Arriba [21] were available from the Genomic Data Commons Data Portal. These samples typically have much fewer gene fusions and the range of medium to high confidence fusion transcripts from Arriba with at least 3 supporting discordant reads pairs at the RNA level was 0–9 for DLBC (mean 3, n = 7) and 0–7 for LAML (mean 1.7, n = 31, see Additional file 1: Figure S2). Recurrent fusions included *PML-RARA* and tandem duplication of *KMT2A* in LAML samples. A list of all validated fusions in the TCGA DLBC and LAML samples is included in Additional file 3.

### Comparison of our pipeline to established tools

**Fig. 4:**
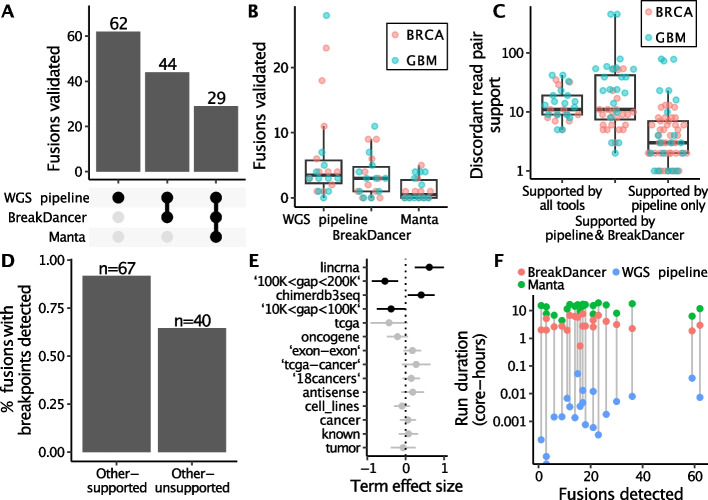
Comparison of our WGS fusion validation pipeline to other tools. Our pipeline is sensitive, confirming the presence of 62 fusions that were not detected by BreakDancer or Manta (**A**). Every fusion event validated by BreakDancer or Manta was also detected by our pipeline in both BRCA and GBM samples (**B**). Samples validated by our pipeline alone had fewer discordant read pairs supporting each fusion event on average, although we were able to several highly supported events in GBM that were missed by the other tools (**C**). Fusions that were also detected by Manta or BreakDancer were more likely to be further supported by a genomic breakpoint (**D**). Our pipeline was better at validating fusions between close genes, and the other tools validated a disproportionate number of lincRNA fusions and known fusions listed in the ChimerDB 3.0 database (**E**). Our pipeline was substantially faster to run than the other tools, with each sample running in a matter of seconds as opposed to hours (**F**)

We compared the performance of our pipeline for BRCA and GBM tumors to two widely used tools for detection of structural variants in WGS data, Manta (v. 1.0.3) and BreakDancer (v. 1.4.5-4e44b43). While these tools are primarily designed for the detection of structural variants in WGS data rather than specifically targeting fusion events, they have been widely used in fusion detection studies and have demonstrated utility in identifying fusion events from WGS data [22, 23]. Our pipeline consistently outperformed both Manta and BreakDancer in every sample. All fusions that were validated by Manta or BreakDancer were also detected and validated by our pipeline, indicating that our pipeline is at least as sensitive as these established tools (Fig. 4A, B). Fusions that were detected only by our pipeline had on average a lower number of discordant read pairs supporting the fusion. The distribution of supporting read pairs for validated fusions in shown in Additional file 1: Figure S1B. Overall, only 63 out of the 135 fusions validated by our pipeline could be validated by either of the other tools, and no fusions were detected by Manta that were not also detected by BreakDancer (Fig. 4C). Fusions that were validated by either of the other tools were more likely to have a genomic breakpoint identified in either fusion partner (*p* = 2.3 × 10^–4^, Chi-square test) (Fig. 4D).

Next, we looked at whether fusions that could only be detected by our pipeline had any characteristics that set them apart from other fusions. We did not find differences in the predicted effects of the fusion (i.e. whether the fusion partners were in-frame, promoter-swapping, etc.) between fusions validated by our tool vs other tools. Our pipeline validated more fusion events between close genes compared to the other tools, showing an enrichment in the FusionCatcher description tags 10 K < gap < 100 K and 100 K < gap < 200 K when modeled in a linear regression (*p* = 0.04 and *p* = 0.002, respectively, F-test). Similarly, Manta and BreakDancer were more likely to validate fusions involving long intergenic non-coding RNAs (lincRNAs) and events listed in the ChimerDB 3.0 database [24] (*p* = 0.001 and *p* = 0.02 respectively, F-test, Fig. 4E).

An additional advantage with our pipeline is that it runs much faster than the other tools, as it does not perform a genome-wide identification of fusion events but instead uses a list of previously detected fusion transcripts. Our pipeline took on average only 26 s to run per sample, as opposed to 3 h 37 min and 12 h 33 min for BreakDancer and Manta, respectively (Fig. 4F).

### Detection of experimentally validated gene fusions

**Fig. 5:**
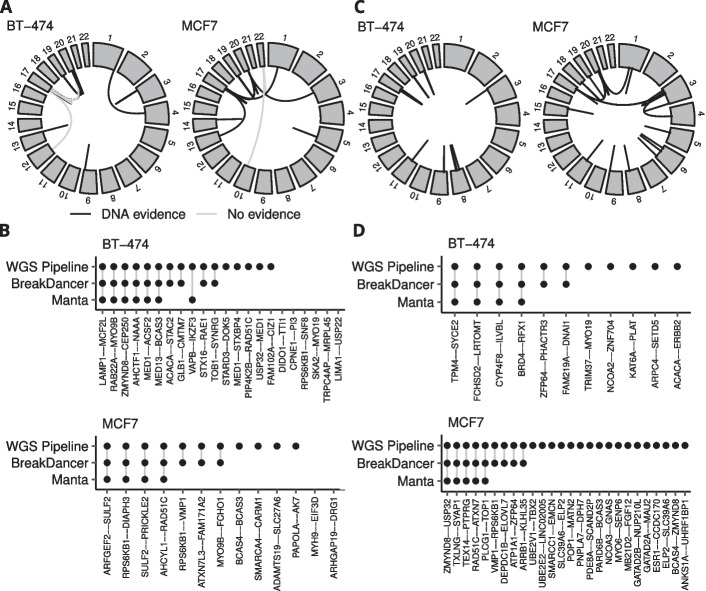
Experimentally validated fusions in BT-474 and MCF7 (**A**). The majority of previously reported fusions in these cell lines were validated by our pipeline, which was more sensitive than both Manta and BreakDancer (**B**). We were able to validate additional fusions at the DNA level using our pipeline (**C**), including several that were also detected by other tools (**D**)

Several studies have identified and experimentally validated gene fusions in common breast cancer cell lines [25–28]. To further evaluate the performance of our pipeline we applied it to a list of previously identified and validated fusions in the breast cancer cell lines BT-474 and MCF7 (Fig. 5A). We compared the results to Manta and BreakDancer and analyzed the overlap of the fusions detected between the different tools. Our fusion validation pipeline was again more sensitive than both Manta and BreakDancer, finding evidence for 27 out of 35 previously reported gene fusion events, including 5 fusions in BT-474 and 4 fusions in MCF7 that were not detected by either of the other tools. Overall, the sensitivity was 0.79 for our pipeline, 0.48 for BreakDancer and 0.31 for Manta (Fig. 5B).

To produce a more complete view of the fusion landscape of these two cell lines, we ran FusionCatcher on RNA-Seq data for these cell lines generated by the Cancer Cell Line Encyclopedia (CCLE). After filtering to keep only high-confidence fusions as before, we identified 150 putative fusion transcripts across both cell lines including 120 that were not previously experimentally validated. Among these, we could validate 35 new fusions at the DNA level. Fifteen of them were also found by either Manta or BreakDancer, with both tools displaying similar relative sensitivities as for the experimentally validated fusions (Fig. 5C, D). A list of all validated fusions in BT-474 and MCF7 is included in Additional file 4.

Among the published lists of experimentally validated fusions, we were able to detect all but two at the RNA level using FusionCatcher: LIMA1-USP22 in BT-474 and ARHGAP19-DRG1 in MCF7. This was still the case even after re-running the software using settings for increased sensitivity. We were also unable to validate them at the DNA level with any of the three tools. Three fusion events were flagged by FusionCatcher as having a high likelihood of being false positives; two events were between a gene and its pseudogene (RAB22A-MY09B and ARFGEF2-SULF2) and the third was a fusion between adjacent genes (PAPOLA-AK7). Although these events were flagged by FusionCatcher as being likely false positives, supporting reads were still found at the DNA level.

We also analyzed six cell lines of hematological origin that had known oncogenic driver fusions as well as publicly available RNA-Seq and WGS data (CCRF-CEM, LAMA-84, MOLM-13, MOLT-4, MV4-11, and THP-1). The data was analyzed with FusionCatcher followed by validation using our pipeline. The expected *BCR-ABL1* fusion [29] was detected in LAMA-84 and previously reported *KMT2A* fusions were found in MOLM-13, MV4-11, and THP-1 [30–32] (see Additional File 1: Figure S3 and Additional file 4). Unfortunately the WGS data that was available for CCRF-CEM and MOLT-4 had a sequencing depth below 1X, compared to a range of 21-42X for the other cell lines. At this depth our pipeline could not find any discordant read pairs to support the previously reported *TAL1* fusions [33].

## Discussion

In this study, we have developed a bioinformatic pipeline to validate fusion transcripts detected at the RNA level using matched WGS data. We found that our pipeline was able to detect a significant proportion of highly supported fusion transcripts predicted by FusionCatcher. We used our pipeline to confirm the presence of fusions in TCGA tumor samples, as well as previously validated fusions in the breast cancer cell lines BT-474 and MCF7. Compared to other tools that can be used to detect fusions at the DNA level, our validation pipeline is more sensitive and computationally efficient, suggesting that it is an effective method of validating the presence of fusions in solid tissue tumors. In addition, our pipeline was also able to identify genomic breakpoints in close proximity to discordant read pairs that supported the observed fusion. Information about the exact genomic breakpoint can greatly facilitate experimental validation of identified fusion genes. FusionCatcher was able to detect the presence of nearly all previously experimentally validated gene fusions. We were unable to validate the remaining fusions in WGS data, indicating that those fusion might not be ubiquitous in the cell line they were detected in.

As our pipeline is designed so that only a very limited area of the genome is searched for each observed fusion transcript event, we can afford to use very sensitive methods that would otherwise generate false positives if used on a genome-wide scale. This also allows us to greatly optimize the run-time of the pipeline compared to genome-wide tools, with results generated in a matter of seconds if the list of fusions used as input is small. As our pipeline relies on an input of fusion transcripts, it is unable to detect other forms of structural variants or non-expressed gene fusions. If such results are desired, the use of other tools is recommended.

A potential limitation of our study is that it relies on the presence of discordant read pairs and reads with high-quality soft-clipped ends to validate fusions and detect breakpoints, respectively. This means that we are limited by the sequencing depth of the WGS data in question. However, we have shown that our pipeline is highly sensitive and specific in identifying fusions in solid tissue tumors, and that the number of reads supporting a genomic breakpoint does not correlate with sequencing depth among the analyzed samples. A creative approach to identify genomic breakpoints without available WGS data using intronic reads found in total RNA-Seq data was used in a large study of gene fusions in childhood cancer [34]. The results correlated well with breakpoints found in matched WGS data but were limited by sequencing depth and expression level.

Experimental validation of fusions remains a challenge. In evaluating our pipeline against a list of experimentally validated fusions, we found that there is little overlap between cell line fusions in publications looking at this topic, and in addition the studies missed likely true-positive events. The results of these studies are therefore not a gold standard that can be confidently used to evaluate in-silico tools, which negatively impacts the assessment of sensitivity and/or specificity of any given software.

In summary, our fusion detection pipeline represents a valuable tool for identifying fusions in solid tissue tumors with high sensitivity and specificity. Our approach is useful in the development of personalized cancer therapy and warrants further investigation in larger cohorts of patients with solid tissue tumors. Our pipeline can also be used to gain insight into the mechanisms behind the creation of gene fusions as we can extract the exact genomic breakpoints of fusion events. Working with a list of validated fusion events will help in determining their functions as there will be fewer false-positive fusions generating noise and hindering meaningful biological conclusions.

## Conclusions

We have developed a fast and sensitive pipeline for validation of gene fusions detected by RNA-Seq in matched WGS data.

### Availability and requirements

Project name: WGS fusion validation pipeline.

Project home page: https://github.com/VolundurH/wgs_fusion_pipeline

Operating system(s): Linux.

Programming language: R (≥ 4.0).

Other requirements: None.

License: MIT.

Any restrictions to use by non-academics: None.

### Supplementary Information

**Additional file 1**. Supplementary Figures.

**Additional file 2**. Validated fusions in TCGA BRCA and GBM samples.

**Additional file 3**. Validated fusions in TCGA DLBC and LAML samples.

**Additional file 4**. Validated fusions in cancer cell lines.

## Data Availability

The source code for the fusion WGS validation pipeline is available in the GitHub repository (https://github.com/VolundurH/wgs_fusion_pipeline). In addition, we provide a singularity container with all prerequisite software installed. The datasets analyzed are available from the TCGA Research Network, https://www.cancer.gov/tcga and from the Sequence Read Archive, https://www.ncbi.nlm.nih.gov/sra.

## Abbreviations

bp: Base pairs
BRCA: Breast invasive carcinoma
GBM: Glioblastoma multiforme
kb: Kilobases
lincRNA: Long intergenic non-coding RNA
ncRNA: Non-coding RNA
RNA-Seq: RNA sequencing
TCGA: The Cancer Genome Atlas
WGS: Whole-genome sequencing
