# Supplementary material for: Fast and sensitive validation of fusion transcripts in whole-genome sequencing data
Source: BMC Bioinformatics. 2023 Sep 23;24:359. doi: 10.1186/s12859-023-05489-5 (PMC10518092; doi:10.1186/s12859-023-05489-5)
Supplement: Supplementary file 1 — Additional file 1. Supplementary Figures. [file 12859_2023_5489_MOESM1_ESM.docx]

**Additional file 1**

**Fast and sensitive validation of fusion transcripts in whole-genome sequencing data**

Völundur Hafstað^1^, Jari Häkkinen^1^ and Helena Persson^1*^

**Author affiliations:**

^1^Lund University Cancer Centre, Faculty of Medicine, Department of Clinical Sciences Lund, Oncology, Lund, Sweden

* Corresponding author:

Helena Persson

Lund University

Dept of Clinical Sciences Lund, Oncology

E-mail: [helena.persson@med.lu.se](mailto:helena.persson@med.lu.se)

**Figure S1.** Fragment size distribution for aligned read pairs in the TCG-BRCA samples (A). Number of supporting read pairs for validated fusions in the TCGA BRCA and GBM samples (B).

**Figure S2.** Number of medium- and high-confidence predicted fusion transcripts from Arriba in the TCGA DLBC and LAML cohorts (A). A higher fraction of fusions was validated among the medium- and high-confidence fusion transcript predictions in both cohorts (B). Number of supporting discordant read pairs for validated fusions split by TCGA cohort and Arriba prediction confidence level (C). Number of reads supporting identified genomic breakpoints for each cohort (D) and split by Arriba prediction confidence level (E).

**Figure S3.** Fusion events predicted by FusionCatcher and validated by our pipeline in six cell lines of hematological origin include KMT2A fusions in three cell lines (MV4-11, MOLM-13, THP-1)
